# Spatiotemporal Changes of Cyanobacterial Bloom in Large Shallow Eutrophic Lake Taihu, China

**DOI:** 10.3389/fmicb.2018.00451

**Published:** 2018-03-21

**Authors:** Boqiang Qin, Guijun Yang, Jianrong Ma, Tingfeng Wu, Wei Li, Lizhen Liu, Jianming Deng, Jian Zhou

**Affiliations:** ^1^State Key Laboratory of Lake Science and Environment, Nanjing Institute of Geography and Limnology, Chinese Academy of Sciences, Nanjing, China; ^2^School of Environment and Civil Engineering, Jiangnan University, Wuxi, China; ^3^Chongqing Institute of Green and Intelligent Technology, Chinese Academy of Sciences, Chongqing, China; ^4^Research Center of Poyang Lake, Jiangxi Academy of Sciences, Nanchang, China

**Keywords:** hydrodynamics, cyanobacterial bloom, *Microcystis* colonies, turbulence, wind, Lake Taihu

## Abstract

Lake Taihu is a large shallow eutrophic lake with frequent recurrence of cyanobacterial bloom which has high variable distribution in space and time. Based on the field observations and remote sensing monitoring of cyanobacterial bloom occurrence, in conjunction with laboratory controlled experiments of mixing effects on large colony formation and colonies upward moving velocity measurements, it is found that the small or moderate wind-induced disturbance would increase the colonies size and enable it more easily to overcome the mixing and float to water surface rapidly during post-disturbance. The proposed mechanism of wind induced mixing on cyanobacterial colony enlargement is associated with the presence of the extracellular polysaccharide (EPS) which increased the size and buoyancy of cyanobacteria colonies and promote the colonies aggregate at the water surface to form bloom. Both the vertical movement and horizontal migration of cyanobacterial colonies were controlled by the wind induced hydrodynamics. Because of the high variation of wind and current coupling with the large cyanobacterial colony formation make the bloom occurrence as highly mutable in space and time. This physical factor determining cyanobacterial bloom formation in the large shallow lake differ from the previously documented light-mediated bloom formation dynamics.

**Significance Statement**:

(1) Use of various tools such as remote sensing, field *in situ* observation and laboratory controlled experiments to characterize the cyanobacterial bloom occurrence related to the hydrodynamic actions in this large shallow and eutrophic lake.
(2) Moderate or small disturbance will promote colony enlargement through cell/colony aggregation due to the presence of extracellular polysaccharide (EPS). Cells/colonies aggregation increase the colony buoyance and speed up the surface visible cyanobacterial bloom formation.
(3) The large variation of wind-induced disturbance interacting with the buoyant colony aggregation determine the high spatiotemporal heterogeneity of cyanobacterial bloom distribution in this large shallow and eutrophic lake.

## Introduction

Most fresh water lakes in China are shallow and generally polluted with nutrient over-enrichment (Liu and Yang, 2012). One direct consequence of eutrophication is an increase in the occurrence of cyanobacterial blooms (Huisman et al., 2005). The majority of bloom-forming algal species are toxic (Chorus and Bartram, 1999) hence threatening drinking water safety (Guo, 2007; Qin et al., 2010) and disrupting the food chain (Micheli, 1999). Furthermore, decaying blooms may cause anaerobic conditions, which adversely affect aquatic life (Diaz and Rosenberg, 2008), resulting in odorous “black water” conditons (Qin et al., 2015; Zhang et al., 2016), accompanied by biodiversity decreases (Vonlanthen et al., 2012).

The formation of a cyanobacterial bloom is determined by sufficient algal biomass, cellular buoyancy and hydrodynamic conditions (Reynolds, 2006). Cyanobacterial biomass is determined by nutrient concentration (Schindler, 1974; Smith et al., 1999), water temperatures (Paerl et al., 2011; Deng et al., 2014), underwater light (Foy et al., 1976; Oliver, 1994; Reynolds, 2006). The physical conditions such as mixing intensity affect the bloom spatiotemporal distribution (Cao et al., 2006; Hunter et al., 2008; Wu et al., 2013). With absence of disturbance, cellular buoyancy depends on the size, shape, and density according to Stokes’s law (Oliver, 1994). A positive buoyancy causes cyanobacteria to float upward and aggregate at the water surface, forming dense surface blooms when disturbance is weak or absent (Ibelings, 1996; Wallace and Hamilton, 1999; Paerl et al., 2011). The formation of blooms allows cyanobacteria to gain more access to light than other species of algae, thereby suppressing the growth of competing algal taxa (Moisander et al., 2002; Huisman et al., 2004; Paerl et al., 2011). In contrast, during strong mixing, turbulence can entrain cyanobacteria below the euphotic zone where they experience sub-optimal light conditions (Huisman and Weissing, 1994; Wallace and Hamilton, 1999; Paerl et al., 2011). In some lakes, such intensive mixing can cause the dominant algal species shift from cyanobacteria to green algae and diatoms (Reynolds et al., 1983; Moreno-Ostos et al., 2009; Visser et al., 2016).

But the cyanobacteria bloom occurrence in Lake Taihu is quite different. As a large shallow and eutrophic lake, the cyanobacteria bloom occurrence is characterized by rapid changes in situation and extent, which is likely coming and going without a trace. The duration of the cyanobacteria bloom often lasted a few hours (**Supplementary Table S1** and **Supplementary Figure S1**), similar phenomenon was found in Lake Chao, the fifth largest freshwater lake in China (with area 825 km^2^ and mean depth ∼4 m) (**Supplementary Table S2**). It was, therefore, very difficult to monitor, track, simulate and predict. The bloom forming species in Lake Taihu is buoyant *Microcystis* which can account for 90% of total phytoplankton biomass during summer (Chen et al., 2003). Meanwhile, the cyanobacteria cells present in field in a form of large colonies (a number of cells stick together) which take an overall majority (Wu and Kong, 2009). It is supposed that the extracellular polysaccharide (EPS) supports aggregation of cells (Xu et al., 2013; Li et al., 2013). Because the large colonies have high buoyancy so as to sustain the colonies floating at water surface, the bloom forming cells/colonies are likely more susceptible to wind induced water movement. Here we hypothesized that the high spatiotemporal variation of cyanobacterial bloom in this large shallow lake are caused by wind-induced hydrodynamic mixture and advection associated with the presence of EPS. In this study, we examined the wind induced physical effects on the cyanobacterial bloom formation via field observations and laboratory controlled experiments. The purpose of this paper is to address the dynamics of bloom change in space and time in Lake Taihu, particularly the wind-induced mixing effects on the formation of cyanobacterial colonies and dense aggregation bloom at water surface.

## Data and Methods

### The Study Site

Lake Taihu, the third largest freshwater lake in China, is located in the Yangtze River Delta region. It’s area is 2338 km^2^, mean depth 2 m, and the maximum depth is no more than 3 m with flat-bottomed bathymetry. There is no persistent stratification during summertime. Its eutrophication began in the 1980s (Qin et al., 2007), and cyanobacteria bloom coverage has gradually extended from Meiliang Bay (Chen et al., 2003) to the entire northwest zone of the lake. The bloom-forming species is high buoyant *Microcystis* which account for overwhelming majority during summer (Chen et al., 2003). The nutrient enrichment combined with warm spring resulted in the earlier occurrecne of intensive cyanobacterial bloom which caused the drinking water crisis in late May 2007 (Qin et al., 2010). With a series of pollution control measures implemented since 2007 (Stone, 2011), the water quality has not further deteriorated, but the notorious harmful cyanobacterial blooms still persist and the bloom induced anaerobic “black water” recur throughout summer in this lake (Zhang et al., 2016).

### *In Situ* Observation of *Microcystis* Colony Formation and Cyanobacterial Bloom Occurrence

**FIGURE 1:**
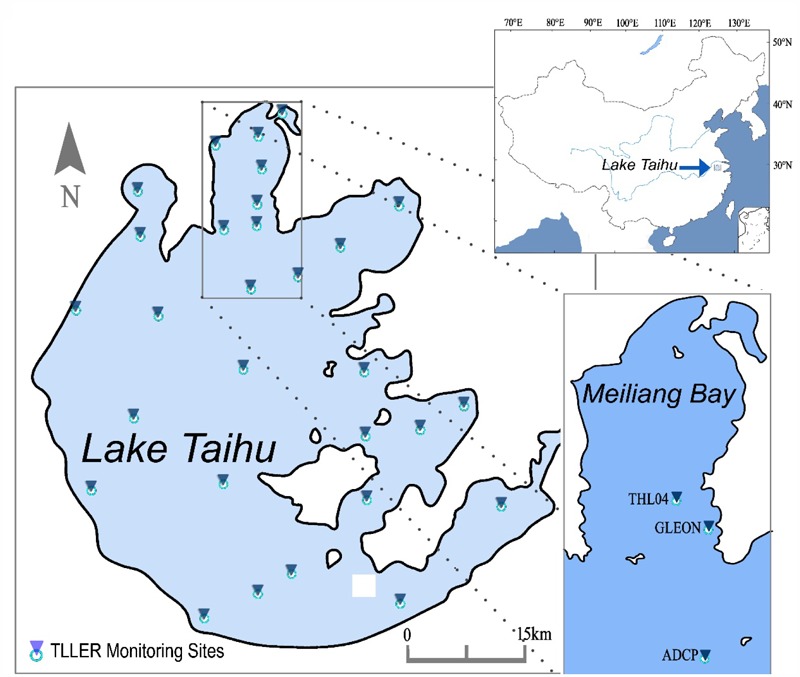
Location of Lake Taihu, Taihu Laboratory for Lake Ecosystem Research (TLLER) and Wuguishan Islet for Acoustic Doppler Current Profiler (ADCP) and multi-parameter water quality instrument deploying.

To investigate the impact of wind-induced mixing on the cyanobacteria bloom occurrence, multi-parameter monitoring was conducted synchronously during both Typhoon Morakot (August 12–14, 2009) and Typhoon Soulik (July 12–13, 2013). Instruments to record wind velocity and direction, at a frequency of every 10 min, were moored at the pier end in Taihu Laboratory for Lake Ecosystem Research (TLLER), Chinese Academy of Sciences (CAS) nearby the Meiliang Bay in Lake Taihu (**Figure 1**). We were particularly interested in the vertical migration speed of *Microcystis* colonies, therefore, an Acoustic Doppler Current Profilers (ADCP) was deployed at the bottom of the water column near Wuguishan Islet to record three-dimensional current velocity (**Figure 1**). At the same site, multi-function water quality sensors (YSI6600) were deployed near the water surface (ca. 20 cm under surface) and the bottom (50 cm above bottom of lake). The sensors of water quality parameters, including pH, conductivity, turbidity, were recording with a 10 min resolution, while the chlorophyll-a concentration was recorded every 30 min by *in vivo* fluorescent light detectors which need to be adjusted before deploying.

Additionally, cyanobacteria colonies were sampled during typhoon Soulik at the end of pier in TLLER (**Figure 1**). Samples were taken at noon of July 12 and 13, 2013, followed by a period during which the sampling interval was 12 h from the midnight of July 13 to the noon of July 15, 2013. Samples were also collected around midnight of July 15 and in the morning of July 16, 2013. The depths of sampling included the bottom layer (0.1 m above sediments), middle layer (1.2 m above sediments) and surface layer (2 m above sediments). The colony size of *Microcystis* were counted from at least 30 colonies (>100 cells) with microscopy (Nikon E200). Microscopic examination showed that the dominant phytoplankton species were *Microcystis aeruginosa, M. flos-aquae*, and *M. wesenbergii* during observations, and the proportion of *Microcystis* accounted for more than 90% of total phytoplankton biomass. For convenience, we took the concentration of Chl *a* to represent *Microcystis* biomass.

### *In Situ* Experiment of Mixing Induced by Wind-Wave on Colony Size of *Microcystis* in Lake Taihu

In order to examine whether the disturbance by wind is significant to promote the formation of *Microcystis* colony, an experiment was carried out under the pier in TLLER on September 23, 2017. Some *M. aeruginosa* and *M. flos-aquae* colonies separated from Meiliang Bay, Lake Taihu, were cultured with BG11 (TN = 50 mg/L, TP = 2.5 mg/L) for 2 months followed by extended 2 months culturing with modified BG11 (TN = 10 mg/L, TP = 0.5 mg/L); and a samples with single cell, unicells and small colonies of pure *Microcystis* were obtained. About 500 ml cultured pure *M. aeruginosa* (1.59 × 10^5^ cells/mL) and *M. flos-aquae* (1.59 × 10^5^ cells/mL) were transferred to pre-cleaned plastic bottle. Three 500 ml bottles were fixed to iron pile of the nearby as control of no wind induced disturbance; while the other three bottles as treatments, tethered to cement pile by rope, swing with the water movements induced by wind (**Supplementary Figure S2**). This experiment took 48 h. The wind speeds were measured every 1 h during experiment. *Microcystis* biomass, *Microcystis* colony size and EPS concentration in the bottles were measured at-beginning and at-end of experiment. The content of soluble extracellular polysaccharide (sEPS) and bound extracellular polysaccharide (bEPS) were quantified spectrophotometrically by the anthrone method (Herbert et al., 1971). Samples of *M. aeruginosa* (5 mL) and *M. flos-aquae* (5 mL) were preserved with Lugol’s iodine solution. After the *M. aeruginosa* and *M. flos-aquae* settled for 48 h, samples of *M. aeruginosa* (5 mL) and *M. flos-aquae* were concentrated (1 mL). Then *M. aeruginosa* and *M. flos-aquae* colony size was measured (400× magnification) with a Nikon E200 microscope and QCapture pro. software. The mean colony size of *M. aeruginosa* and *M. flos-aquae* were measured under microscopy with at least 50 colonies.

### Effects of *in Situ* Simulated Mixing on Colony Size of *Microcystis* in the Lake Taihu

For further investigating the influence of disturbance on the formation of *Microcystis* colony, a simulated perturbation experiment was carried out in July in TLLER. Similarly, we took six big plastic barrels (three for control and three for treatment) and each barrel has 90 cm in diameter and 80 cm in height (**Supplementary Figure S3**). All barrels were filled with water containing *Microcystis* colonies taken from Meiliang Bay, Lake Taihu, to the depth of 60 cm. Total nitrogen (TN) and total phosphorus (TP) concentrations in barrels were adjusted with addition of NaNO_3_ and K_2_HPO_4_ 3H_2_O to 5 mg/L for TN and 0.25 mg/L for TP which were almost two times of TN and TP concentration in Meiliang Bay, Lake Taihu. Six barrels were cultured in static condition during the first 5 days. During this period, samples were taken at 10:00 am every morning from two layers at the surface (50 cm above bottom) and the bottom (0 cm above bottom). From the 6th day, the treatments were imposed with wave produced by mini wave action generators (WP-60, Triopo Electric Co. Ltd.) which were deployed at 10 cm below the surface. The wave was produced with a frequency of 1 Hz and the maximum wave height was 5 cm (**Supplementary Figure S3**). Wave disturbances were lasting for 24 h. During the disturbance period, water samples were taken at 3, 6, 12, and 24 h after the beginning; and during the post-disturbance (wave generator stopped to work but the wave effects still existed), samples were taken at 3, 6, 12, 24, and 48 h. Sample analysis for *Microcystis* biomass and colony size and the concentrations of EPS were determined as described in Section “*In Situ* Experiment of Mixing Induced by Wind-Wave on Colony Size of *Microcystis* in Lake Taihu.” The concentrations of Chl *a*, TN and TP were determined by spectrophotometry. Microscopic examination showed that *Microcystis* was the overwhelming dominant species during experiment.

### Effects of Simulated Mixing on the Colony Size of *Microcystis* in Laboratory

Single colony of *M. aeruginosa* and *M. flos-aquae* were isolated from lake water in Meiliang Bay (dominated by *Microcystis* bloom) in Lake Taihu and were cultured with BG-11 medium. Two months later, unialgal cultures of *M. aeruginosa* and *M. flos-aquae* were transferred to modified BG-11 medium (TN = 50 mg/L, TP = 2.5 mg/L), respectively. Until the beginning of experiment, *M. aeruginosa* and *M. flos-aquae*, present as single-cells, paired-cells and small colonies, were cultured in modified BG-11 media for 5 months. At the beginning of the experiment, 150 mL *M. aeruginosa* (4.83 × 10^6^ cells/mL) and *M. flos-aquae* (1.78 × 10^6^ cells/mL) were transferred to 500 mL Erlenmeyer flasks containing 200 mL modified BG-11 medium. Considering the velocities (0.005–0.077 m/s) of Lake Taihu (Zhou, 2016), five different mixing intensities were designed as following: 0, 50, 100, 200, and 400 rpm, which were corresponding to velocities of 0, 0.16, 0.32, 0.64, and 1.28 m/s (Camacho et al., 2007; Guadayol et al., 2009; Gallardo Rodríguez et al., 2009), respectively. The control had three 500 ml flasks with shaking intensity of 0 rpm. Each treatment also had three 500 ml flasks (triplicate) placed on shaking incubator. There were four shaking incubators with shaking intensities of 50, 100, 200, and 400 rpm. The shaking experiment lasted for 24 h at 25°C and cool/white fluorescent lights at an intensity of 40.5 mol m^-2^ s^-1^ with a light-dark cycle of 12:12 h. The total concentration of nitrogen and phosphorus in all treatments and control were maintained about 50 and 2.5 mg/L, respectively. Samples were taken before and after the experiment. The measurements of colony size, biomass of *M. aeruginosa*, concentrations of Chl *a*, EPS, TN, and TP were the same as described in Section “*In Situ* Experiment of Mixing Induced by Wind-Wave on Colony Size of *Microcystis* in Lake Taihu” above.

### Measurements of Vertical Movement Velocity for Different Size Cyanobacteria Colonies

Large size colony has high buoyancy to overcome the mixing and gravity to float at water surface and form visible bloom. To measure the upward movement velocity of different size *Microcystis* colonies, the field samples of *Microcystis* were taken with net filtering (mesh sizes were 425, 100, 64, and 20 μm) to obtain five different size classes of *Microcystis* colonies (>425 μm, 100–425 μm, 64–100 μm, 20–64 μm, and <20 μm). For each colony size group, the cell numbers and proportion to all colonies were estimated before the experiment. A plexiglass column (1 m in height and 0.1 m in diameter) was used to measure the upward movement velocity of different size colonies. There was an inlet for injection at the column bottom and five sampling outlets distributed along the column every 0.2 m. The column was filled with distilled water. Different size colony samples were injected at the bottom. After 1 (>425 μm), 3 (100–425 μm), and 10 min (<100 μm), samples were taken from 5 sampling outlets simultaneously and the density of algae was counted with microscope. Five different size *Microcystis* colonies were injected into the columns separately. Each colony size experiment had triplicate.

The floating velocity of different size colonies was calculated as follows:

(1) $$Floating\;velocity\;=\;\frac{\sum \left(v_{\text{i}}\times B_{\text{i}}\right)}{B_{\text{t}}}$$

where *B*_t_ (the total biomass) equal to the product of injection density and injection volume, *B*_i_ (the biomass of each outlet) was calculated by the cell density of each outlet by 1/6 of the volume of plexiglass column, *v*_i_ (floating velocity of each outlet) were calculated by the height of each sampling outlet divided by experimental time.

### Remote Sensing Technique to Monitor Cyanobacteria Bloom Intensity Changes in Time and Space

Remote sensing about the cyanobacterial bloom area were used to investigate the cyanobacterial bloom changes in space. The lowest cyanobacteria biomass at which the bloom could be detected by satellite was equivalent to the Chl *a* concentration around ∼10^1^ mg/m^3^. All the remote sensing images of cyanobacteria bloom were obtained from NASA^1^. The interpretation of MODIS images was according to the re-projection and geometric correction via ERDAS 9.1, and the information of blooms was retrieved by means of the ratio of near-infrared and red spectrum (Ma et al., 2008; Duan et al., 2009).

## Results

### *In Situ* Field Monitoring of Cyanobacteria Bloom During Typhoon Morakot

**FIGURE 2:**
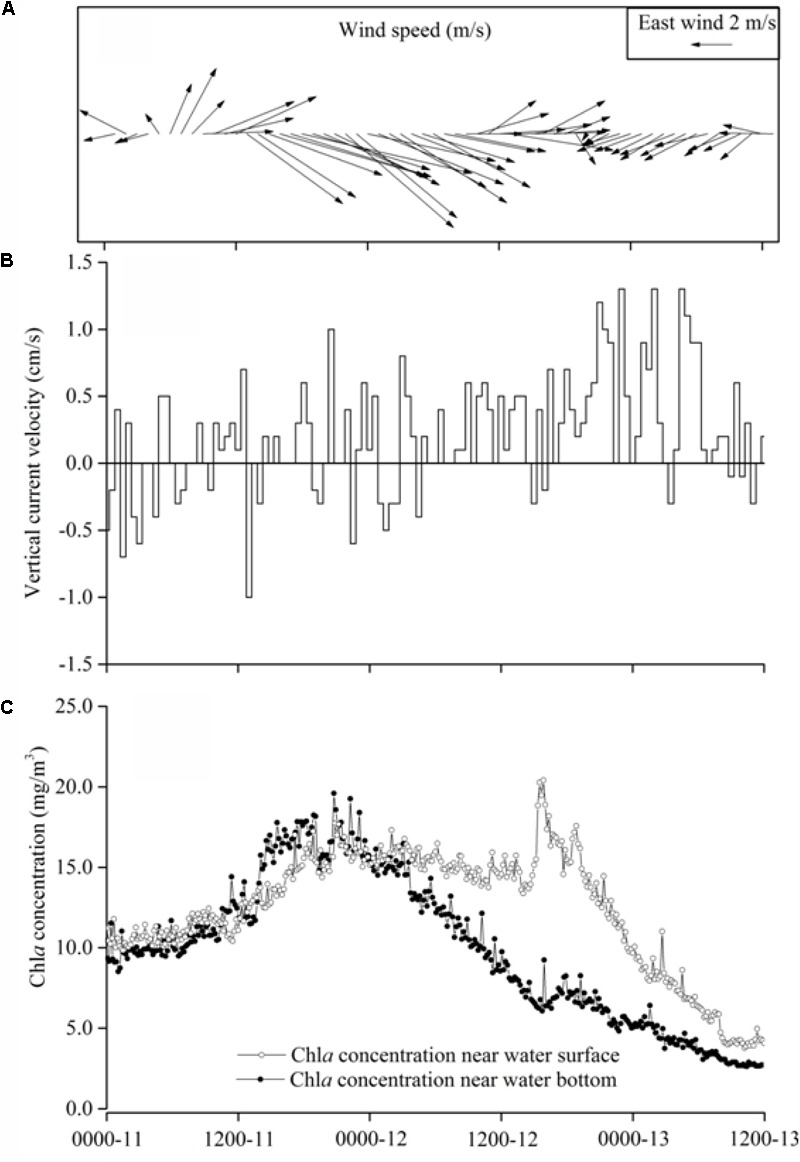
Observations during Typhoon Morakot in August 12–13, 2009 in Lake Taihu. **(A)** The wind speed and wind direction. **(B)** Speed of suspended particulate matter (cyanobacterial colonies) moving up and down recorded by ADCP. **(C)** Chlorophyll-a concentration at the surface layer and the bottom layer.

Lake Taihu basin and delta of Changjiang River (Yangtze River) often experience typhoon during summer. These typhoons normally formed in the tropical or subtropical Pacific Ocean and moved westward or northwestward and influenced the eastern China coastal zone. Typhoon Morakot passed Lake Taihu during August 11–14, 2009. Its effects on the lake started in the early morning of August 11. The dominant wind directions were westerly and northwesterly and the maximum wind speed occurred in the evening of August 11 at 11.8 m s^-1^ (**Figure 2A**), then the wind speed gradually dropped until clam condition in the evening of August 12, while the wind direction shifted to east and northeast. During the typhoon, the near-surface vertical current velocity measured by Acoustic Doppler Current Meter (ADCP), was typically mixing with up and down movement and a maximum velocity was about 0.5 cm/s (**Figure 2B**) to correspond to the high wind speed. After the typhoon, the vertical direction current turned to be upward uniformly and the maximum velocity reached 1.0 cm/s (**Figure 2B**). Because of the measured vertical velocity representing the moving speed of particulate matter in the water column (Holdaway et al., 1999). Thus the measured vertical velocity actually indicated the cyanobacteria colonies moving speed in the water column. *In situ* observation after the typhoon showed that the algal cells/colonies generally ascended to the water surface, instead of sinking, representing that cyanobacteria cells or colonies moving upward was driven by the buoyancy.

Interestingly chlorophyll-a concentrations differed between the layers at near-bottom and near-surface during the post-typhoon. During the typhoon peak period (06:00 h 11 to 06:00 h of August 12), the chlorophyll-a concentrations was almost identical in the water column, suggesting well-mixed conditions during this period (**Figure 2C**). However, after the typhoon, the chlorophyll-a concentration at near-surface layer was increased and that near the bottom layer declined (**Figure 2C**), indicating that cyanobacteria cells/colonies in water column started to differentiate and aggregate at the water surface, which probably a precursor of the cyanobacteria bloom occurrence. Remote sensing images in the morning of August 12, 2009 (during Typhoon Morakot) showed the area of the bloom was only 92 km^2^ (**Supplementary Figure S4A**), which rapidly expanded to 391 km^2^ in the morning of August 13 (**Supplementary Figure S4B**).

### Field Observation of Impact of Typhoon Soulik on Cyanobacteria Colony Formation

**FIGURE 3:**
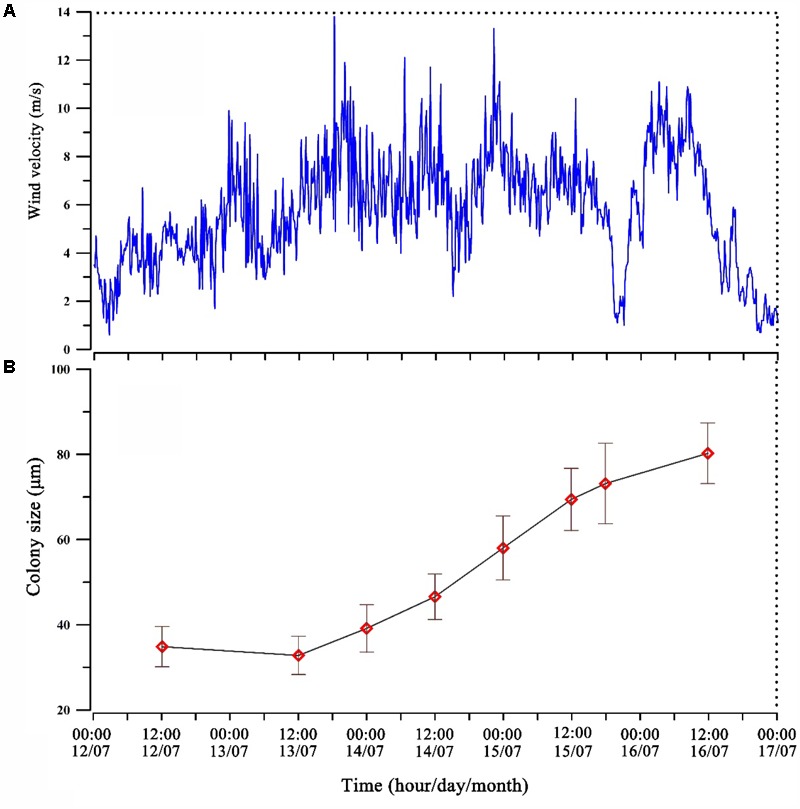
Wind velocity **(A)** and average colony size of *Microcystis* spp. changes in the water column **(B)** before and after the Typhoon Soulik.

Typhoon Soulik affected Lake Taihu during July 12–17, 2013. The period could be divided into three stages according to the wind speed. The pre-typhoon period (00:00–22:00 of July 12), the wind speed increased gradually and average wind speed was 3.82 m s^-1^ (**Figure 3A**). During the typhoon period (from 22: 00 h, 12–18:00 h, July 15) when a high wind speed persisted with average of 6.63 m s^-1^ and a maximum speed of 13.8 m s^-1^ (at 18:00 h, July 13) (**Figure 3A**). The post-typhoon period (18:00 h, 15-00:00 h, July 17) had an average wind speed of 5.26 m s^-1^ which gradually reduced to 1 m s^-1^ near the end of the typhoon (**Figure 3A**). *In situ* measurements revealed that the mean size of *Microcystis* colonies in the water column increased from 32.8 to 69.4 μm within 48 h (**Figure 3B**).

At the same time, remote sensing satellite images showed that little of the cyanobacteria blooms could be seen in the pelagic area of the lake during the Soulik Typhoon peak period from July 12 to July 14; however, around noon of July 17, after the typhoon, blooms occurred with coverage of 288 km^2^ (**Supplementary Figure S5**).

### *In Situ* Experiment of Mixing Induced by Wind-Wave on *Microcystis* Colony Formation in Lake Taihu

**FIGURE 4:**
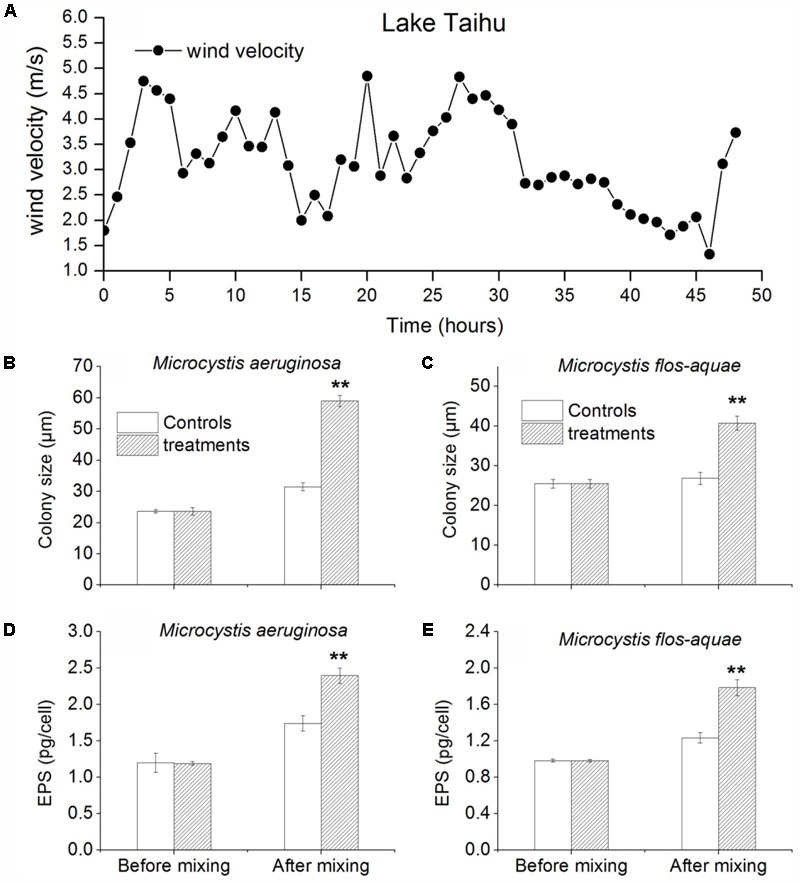
*In situ* experiments about mixing effect on colony size and EPS (extracellular polysaccharide (EPS)) concentrations, and colony size of *Microcystis* at the pier end of TLLER. **(A)** Wind speed during experiment. **(B)** Colony size of *Microcystis aeruginosa* in control and treatment. **(C)** Colony size of *M. flos-aquae* in control and treatment. **(D)** EPS concentration of *M. aeruginosa* in control and treatment. **(E)** EPS concentration of *M. flos-aquae* in control and treatment; error bars was indicated by ± SD ^∗^*p* < 0.05, ^∗∗^*p* < 0.01, *n* = 3).

During this experiment, the mean wind speed was 3.15 m/s (range 1.33–4.83 m/s) (**Figure 4A**). In the treatments (the bottles were not fixed), the colony size of *M. aeruginosa* and *M. flos-aquae* increased quickly after mixing induced by wind-wave for 48 h. The colony size of *M. aeruginosa* in controls (the bottles were fixed) and treatments were 23.6 (±1.2) and 59.0 (±1.8) μm, respectively (**Figure 4B**). The colony size of *M. flos-aquae* in controls and treatments were 25.5 (±1.1) and 40.8 (±1.7) μm, respectively (**Figure 4C**). ANOVA analysis showed that the difference of *M. aeruginosa* and *M. flos-aquae* colony sizes between control and treatment were significantly (*p* < 0.01) (**Figures 4B,C**). Also, ANOVA analysis showed that the EPS concentrations of *M. aeruginosa* and *M. flos-aquae* in treatments were significantly higher than that in controls (*p* < 0.01) (**Figures 4D,E**).

### Effects of *in Situ* Simulative Mixing on Colony Size of *Microcystis* in the Lake Taihu

**FIGURE 5:**
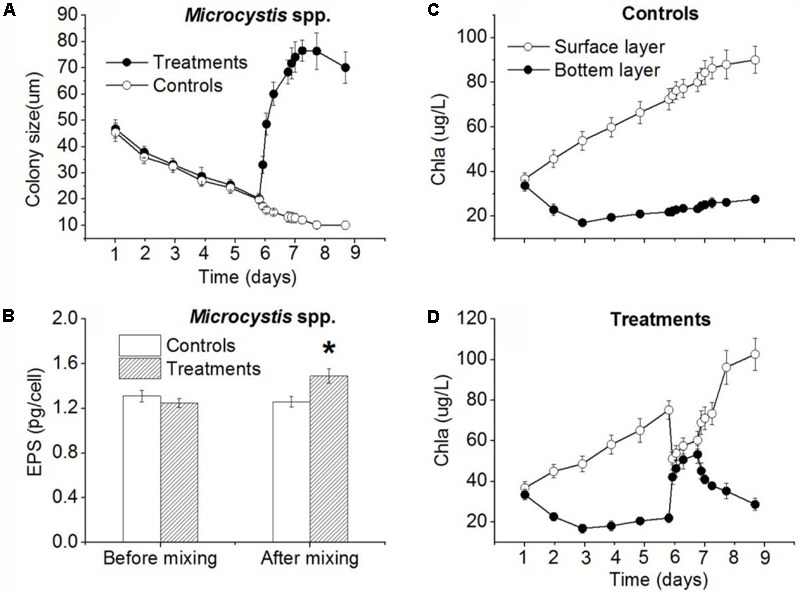
Simulation of mixing effect on *Microcystis* spp. colony size, EPS concentration and vertical distribution of Chl *a* in plastic barrels in TLLER. **(A)** Colony size in control and treatment (including 24-h disturbance during experiment) change with time. **(B)** EPS concentration in control and treatment (including 24-h disturbance during experiment) change with time. **(C)** Surface and bottom Chl *a* concentration in control change with time. **(D)** Surface and bottom Chl *a* concentration in treatment (including 24-h disturbance during experiment) change with time; error bars was indicated by ±SD (^∗^*p* < 0.05, ^∗∗^*p* < 0.01, *n* = 3).

Before the disturbance, the *Microcystis* spp. colony size in the control and treatment decreased gradually (**Figure 5A**). To the 6th day when the disturbance experiment started, the size of *Microcystis* in the control and treatment had reduced from 46.8 μm and 45.4 μm to 19.8 μm and 20.2 μm, respectively. During the disturbance experimental period, the mean *Microcystis* colony size decreased continuously in control while it increased rapidly to 68.4 μm after 24 h disturbance (**Figure 5A**). At the end of experiment (48 h after 24-h disturbance), the average colony size in treatment was 70.1 μm, while the mean colony size in control was 12.6 μm (**Figure 5A**). The colony size difference between control and treatment was significant (*p* < 0.01). After 24-h disturbance, the EPS concentration in the control and the treatment were 1.26 and 1.49 ng/cell, respectively, which was significantly different (*p* < 0.05) (**Figure 5B**). Before the experiment, the initial averaged Chl *a* concentration at the surface and the bottom in the experimental barrels were 36.8 and 33.4 μg/L (**Figure 5C**), respectively. There was no significant difference (*p* > 0.05). During the disturbance period, the surface concentration of Chl *a* decreased sharply to 51.2 μg/L while the Chl *a* concentration at bottom increased rapidly to 42.1 μg/L (**Figure 5D**), which was no significant difference (*p* > 0.05). At the end of the experiment (48 h after 24-h disturbance), the cyanobacterial colonies returned to water surface resulted in Chl *a* concentration of 102.6 μg/L at surface, which was higher than that in control (**Figure 5D**); while the Chl *a* near the bottom was only 28.6 μg/L which differed significantly from that at water surface (*p* < 0.05). The stratification recurred at the end of experiment in treatment (**Figure 5D**).

### Effects of Simulative Mixing on the Colony Size of *Microcystis* in Laboratory

**FIGURE 6:**
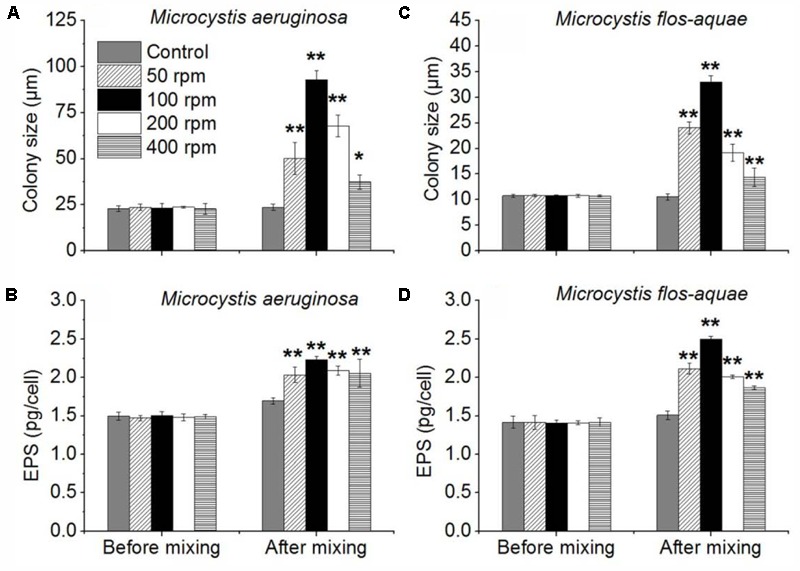
Effects of different mixing intensity (four shaking intensity and control) on colony size and EPS of *Microcystis*. **(A)** Mean *M*. *aeruginosa* colony size of control and 50, 100, 200, and 400 rpm. **(B)** Mean *M*. *aeruginosa* EPS concentrations of control, 50, 100, 200, and 400 rpm. **(C)** Mean *M*. *flos-aquae* colony size of control and 50, 100, 200, and 400 rpm. **(D)** Mean *M*. *flos-aquae* EPS concentrations of control and 50, 100, 200, and 400 rpm; error bars was indicated by ±SD (^∗^*p* < 0.05, ^∗∗^*p* < 0.01, *n* = 3).

In this experiment, the colony size of *M. aeruginosa* and *M. flos-aquae* in 50, 100, 200, and 400 rpm experiments were significantly larger than that in control (*p* < 0.05) (**Figures 6A,C**) after 24-h continuously mixing. The colony size of *M. aeruginosa* in control, 50, 100, 200, and 400 rpm experiments were 23.6 (±1.8), 50.1 (±8.6), 92.9 (±4.8), 67.8 (±10. 9), and 37.3 (±3.9) μm, respectively (**Figure 6A** and **Supplementary Figure S6**). The colony size of *M. flos-aquae* in control, 50, 100, 200, and 400 rpm were 10.6 (±0.6), 24.0 (±1.2), 33.0 (±1.2), 19.2 (±1.6), and 14.4 (±1.8) μm, respectively (**Figure 6C**). Both *Microcystis* species showed an increase firstly with the intensity of mixing followed by a decrease when the mixing intensity larger than 100 rpm (**Figures 6A,C**). In addition, ANOVA analysis indicated that the EPS concentration of *M. aeruginosa* and *M. flos-aquae* in all treatments were significantly higher than that in controls (*p* < 0.01) (**Figures 6B,D**).

### Vertical Migration Rate Measurement for Different Size of Cyanobacterial Colony

**FIGURE 7:**
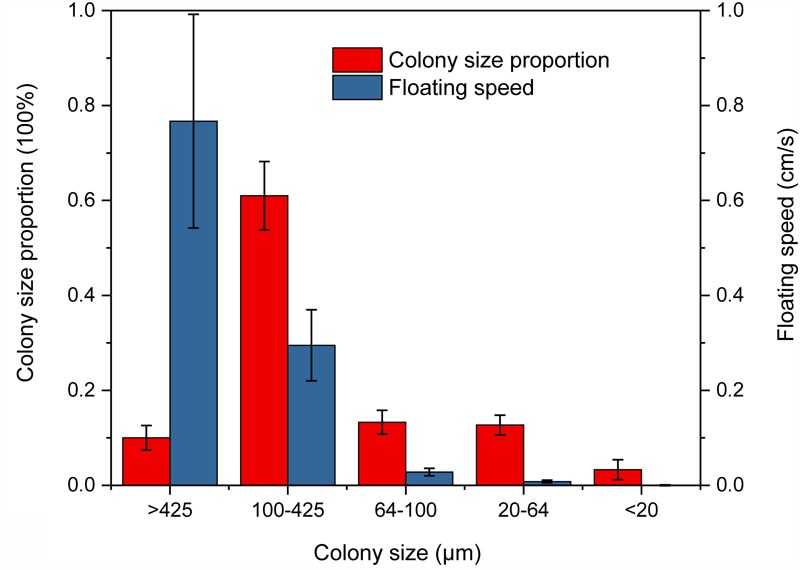
Proportion and floating rate of different *Microcystis* spp. colony sizes in the floating rate measurement experiment.

Experimental results showed that the large size colony (cell count of each colony > 100) accounted for an overwhelming majority in colony size distribution, which was consistent with our field observations (**Figure 7**), and floating rates increased with the colony size. The largest cell colony (>425 μm in diameter) had the floating rate of up to 0.77 ± 0.23 cm s^-1^ (**Supplementary Table S3**). The largest proportion of colony size occurred at colony diameters between 100 and 425 μm with a high floating rate of 0.30 ± 0.08 cm s^-1^ (**Figure 7** and **Supplementary Table S3**), while colony sizes <20 μm were solitary cells or small colonies with floating rates about 0.0002 cm s^-1^ (**Supplementary Table S3**).

## Discussion

**FIGURE 8:**
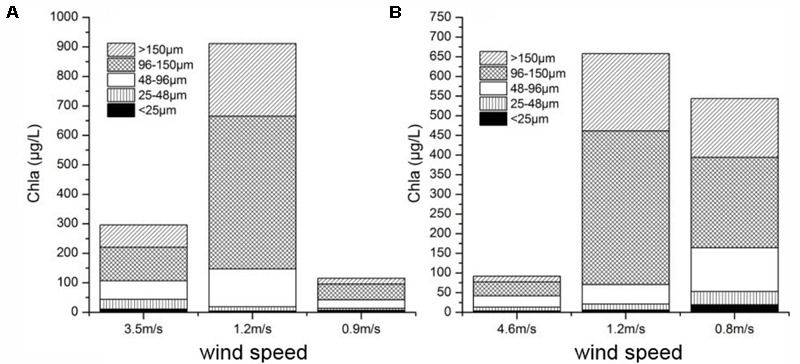
Two *in situ* observations about the colony size composition vs. wind speed at the end of pier in TLLER in **(A)** July 2013 and **(B)** October 2013.

Our field observations and laboratory control experiments suggested that wind induced mixing increased the cyanobacterial colonies size resulted in the increase in colony buoyancy. With the mixing decrease, these large colonies floated upward to surface very quickly to form visible bloom. Lots of researches showed that a surface bloom development will occur only when the cyanobacterial colony buoyancy can overcome the mixing intensity (Denman and Gargett, 1983; Bormans and Condie, 1998; Visser et al., 2016). However, the relationship between the wind-induced hydrodynamic action and cyanobacterial bloom occurrence was not well addressed largely because this relation was simply assumed as a physical process, i.e., the interaction between mixing intensity and the buoyance of cyanobacterial colony. But our field observations and experiments found that the small and moderate physical disturbance will promote the large colony formation and increase the colony buoyance. It can explain why the algal aggregation move upward during the post-typhoons. Field *in situ* observations also found that the surface and bottom Chl *a* concentration were almost same during peak typhoon period indicating that the water column was fully mixed (**Figure 2C**). Meanwhile, the ADCP recorded near-surface vertical movement of colonies was bi-direction. During post-typhoon period, cyanobacterial colonies unifromly ascended to aggregate at water surface (**Figure 2B**) at speed about 0.5 cm/s near surface and 0.3 cm/s near bottom (Wu et al., 2013), which was much higher than previous reported about 10^-4^ m/s (Reynolds et al., 1987) and similar with Visser et al. (1997) documented as 0.7 cm/s for the colony diameter ∼400 μm. This higher vertical speed mainly caused by the buoyancy of large colones of *Microcystis* (Oliver, 1994; Wallace and Hamilton, 1999; Wu and Kong, 2009). According to our investigations in TLLER during July and October of 2013, the large colony (>96 μm) near the water surface took overwhelming majority ranging from 54.4 to 89.2% during different wind speed (**Figure 8**). By our experiment, the ascent rate of big colonies (100–425 μm) was about 1500 times of small colonies (<20 μm) (**Figure 7** and **Supplementary Table S3**), which can explain why the cyanobacterial colonies in the field could congregate quickly at water surface within hours in lake Taihu (**Supplementary Table S1** and **Supplementary Figure S1**). According to the Stoke’s law, the ascent rate of cyanobacterial colonies was determined by the spherical size (indicated as radius) and the density difference between water and *Microcystis* colony (Reynolds et al., 1987). For small colony (<20 μm) of *Microcystis*, almost all cells can get the same amount of light; for large colony (>100 μm) of *Microcystis*, light availability within colony is far less than at the surface of colony, the larger the colony size, the lower the light availability inside the colony, and the lower the colony density. Thus large *Microcystis* colony has low density and high buoyancy. As the large colonies of *Microcystis* have greater buoyancy and higher ascent velocity in the water column compared with solitary cells or small colonies (Xiao et al., 2012), large colonies could move to the water surface in a much shorter time, leading to the “outbreak” of *Microcystis* blooms.

Traditionally, the cyanobacteria cells and colonies aggregation at the water surface results from its metabolism through the gas-vacuolate buoyance adjustment (Reynolds et al., 1987; Oliver, 1994). This accomplished by carbohydrates synthesized during the day increase the cell density to cause cells sink. Conversely carbohydrates are consumed at night, decreasing the cell density and providing positive buoyance to form blooms in the next morning (Visser et al., 1997). If the wind speed were greater than 3 m/s, wind-induced mixing would result the surface visible bloom vanishment (George and Edwards, 1976; Webster and Hutchinson, 1994; Brookes et al., 2003; Wu et al., 2013).

Previous studies already noted that the *M. aeruginosa* colony size can be affected by turbulence-induced aggregation (Naselli-Flores and Barone, 2003; O’Brien et al., 2004; Regel et al., 2004). Our laboratory experiments suggested that persistent and moderate hydrodynamic disturbance can cause aggregation of *M. aeruginosa* and *M. flos-aquae*, and small colonies become larger colonies. But disturbance will not take effect for the solitary cell or unicell on the formation of large colonies via collision (data not shown). This was attributed to the presence of extracellular polysaccharides (EPS) which played a critical role in the large colony formation via collision. EPS are mainly found in mucilage or the cell’s sheath. It can affect the ‘stickiness’ of the cell surface, and further influence large colony formation of *Microcystis* (Yang et al., 2008; Xu et al., 2013; Zhang et al., 2014; Zhu et al., 2014; Pannard et al., 2016). It was documented that the concentration of EPS was significantly higher in colony than in single cell (Li et al., 2013). Our observations suggested that EPS concentration in all mixing experiments were significantly higher than that in controls (**Figures 4–6**). Increasing of EPS after mixing may explain why colony size of *Microcystis* spp. enlarged in all treatments. The large colony formation in this large turbid lake, therefore, was caused by cell-colony or colony-colony aggregation, which was much faster than cell division and proliferation. The spatial distribution of EPS concentration in this lake was highly overlapping the bloom recurrence area (Liu et al., 2014), suggesting that turbulence induced colony aggregation maybe the primary reason for large colony formation and bloom recurrence. In addition, because Lake Taihu is a shallow lake, wind induced disturbance make the sediment resuspension frequently and lead to sharp decrease in light availability underwater, which further result in more gas vesicles of *Microcystis* cells (Walsby et al., 1991). Then *Microcystis* cells or colonies will ascend quickly after the wind halt. But intensive disturbance would disaggregate the large colonies (O’Brien et al., 2004) as it was shown in mixing simulation treatment with rotation rate larger than 100 r/min (**Figures 6A,C**).

For a large shallow lake, quiescent conditions rarely sustain for few hours, thus the cyanobacteria bloom coverage and distribution detected by the satellites or human visions were highly spatiotemporal changeable. The occurrence of cyanobacteria bloom, the location and extent, is the interaction result between colonies buoyancy, mixing intensity and horizontal migration by currents. The location and extent of the bloom, frequently present as a moving patchiness at the surface, can be highly variable. This high variability is originated from the wind-induced waves and currents. Our observation found that the wind speed and direction frequently varied (**Supplementary Figure S7**) because of the influence from rough ground surface, thermal contrast between water-land, etc. Correspondingly, the water current was showing similar variability to winds (**Supplementary Figure S8**). Because of the high variability of winds and water currents in this large shallow lake, it was concluded that the high variability of cyanobacteria blooms originated mostly from the highly changeable wind forcing. Thus the wind induced hydrodynamic actions in Lake Taihu not only determine the cyanobacteria colonies ascent to form surface visible bloom, but also determine the high spatiotemporal heterogeneity of cyanobacterial bloom, and enhance this heterogeneity through the influence on the large colony formation.

Based on the above observations and experiments, we proposed a four-stage of cyanobacteria bloom occurrence in this large shallow eutrophic lake. The first stage is the *Microcystis* cell division and proliferation. During this stage, the cyanobacterial unicells grow and divide, forming small colonies under favorable environmental conditions, such as high temperature, sufficient light and nutrient supply. Small colony will be formed with the secretion of tightly bound extracellular polysaccharides. The second stage is the formation of large cyanobacteria colonies. During this stage, the considerable cells and small-colonies in the water column will increase in size through aggregation. The presence of wind induced disturbance will produce large colonies or disaggregate large colonies. This aggregation and disaggregation process would alternatively happen in the water column until the wind speed declined and a great amount of large colonies formed. The third stage includes the cyanobacteria colonies ascending to form a visible bloom or scum at the water surface when the wind become weak. Moreover, a great amount of larger size colonies formed in water column by mixing and the enhanced buoyancy of larger size colonies allows them to fast aggregate at the water surface. The fourth stage is the onset of more persistent cyanobacteria blooms. In this stage, the blooms drift and aggregate driven by the surface currents and accumulate in the onshore zone, where downward migration is restricted by shallow depth. According to the wind speed measurement in Taihu Laboratory for Lake Ecosystem Research (TLLER), the frequency of calm weather condition (wind speed < 3 m s^-1^) occurs about one sixth of the time. Thus, the wind-induced physical factors such as turbulence may dominate during cyanobacterial bloom occurrence in this large shallow lake.

## Author Contributions

All authors listed have made a substantial, direct and intellectual contribution to the work, and approved it for publication.

## Conflict of Interest Statement

The authors declare that the research was conducted in the absence of any commercial or financial relationships that could be construed as a potential conflict of interest.

## Footnotes

1 http://modis.gsfc.nasa.gov/

## Supplementary Material

FIGURE S1 Variation in cyanobacteria bloom area in Lake Taihu by remote sensing detection at 10:04 h on June 13, 2009 **(A)** and 13:16 h on June 13, 2009 **(B)**.

FIGURE S2 The spot picture of *in situ* experiment of mixing induced by wind-wave on colony size of *Microcystis* in Lake Taihu.

FIGURE S3 The spot picture of *in situ* simulative mixing on colony size of *Microcystis* in the Lake Taihu.

FIGURE S4 The satellite image revealed the cyanobacterial bloom occurrence during post Morak Typhoon. **(A)** Little bloom with area 92 km^2^ was detected at 10:27 am, August 12, 2009, when the typhoon peak had passed. **(B)** Large bloom with area 391 km^2^ was detected at 11:09, August 13, 2009.

FIGURE S5 The satellite images of cyanobacterial bloom during the Typhoon Soulik passed Lake Taihu in July 2013. The left: image of bloom at 13:10 h, July 12, 2013; the middle: image of bloom at 11:15 h, July 14, 2013; the right: image of bloom at 11:50 h, July 17, 2013.

FIGURE S6 The colony pictures of *M. aeruginosa* in controls, 50, 100, 200, and 400 rpm groups after continuing mixing for 24 h in this experiment.

FIGURE S7 The wind velocity and wind direction measurement at the lake center during July 14–August 17, 2015.

FIGURE S8 The water current (about 200 cm above bottom) measurement at the lake center during July 14–August 17, 2015.

TABLE S1 Satellite detected cyanobacterial bloom area changes in a short period in Lake Taihu.

TABLE S2 Satellite detected cyanobacterial bloom area changes in a short period in Lake Chaohu.

TABLE S3 Proportion and floating rate of different colony sizes of *Microcystis*.
