# Supplementary figures and images for: Spatiotemporal Changes of Cyanobacterial Bloom in Large Shallow Eutrophic Lake Taihu, China
Source: Front Microbiol. 2018 Mar 21;9:451. doi: 10.3389/fmicb.2018.00451 (PMC5871682; doi:10.3389/fmicb.2018.00451)

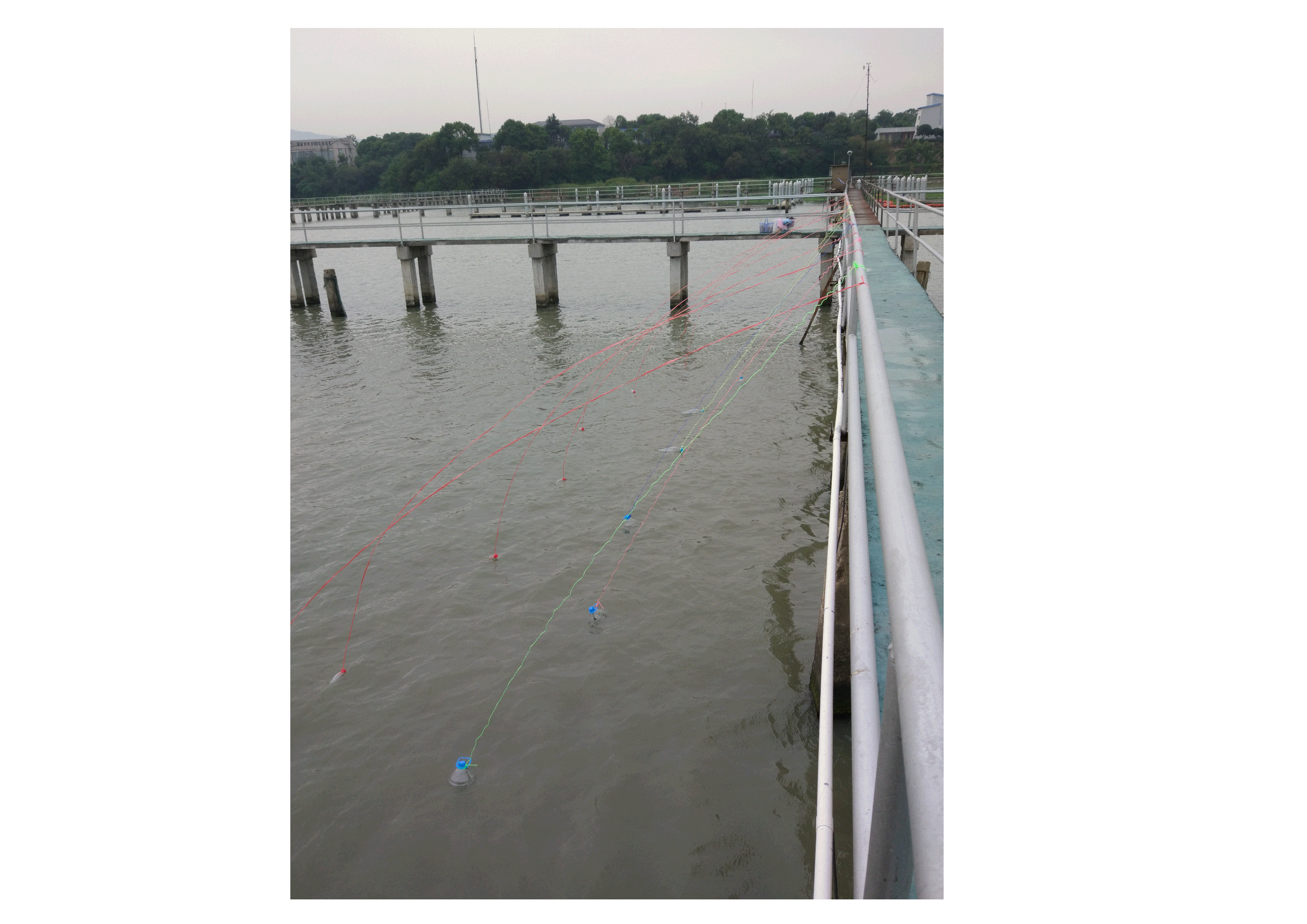

Supplement: FIGURE S1 — Variation in cyanobacteria bloom area in Lake Taihu by remote sensing detection at 10:04 h on June 13, 2009 (A) and 13:16 h on June 13, 2009 (B). [file Image_1.png]

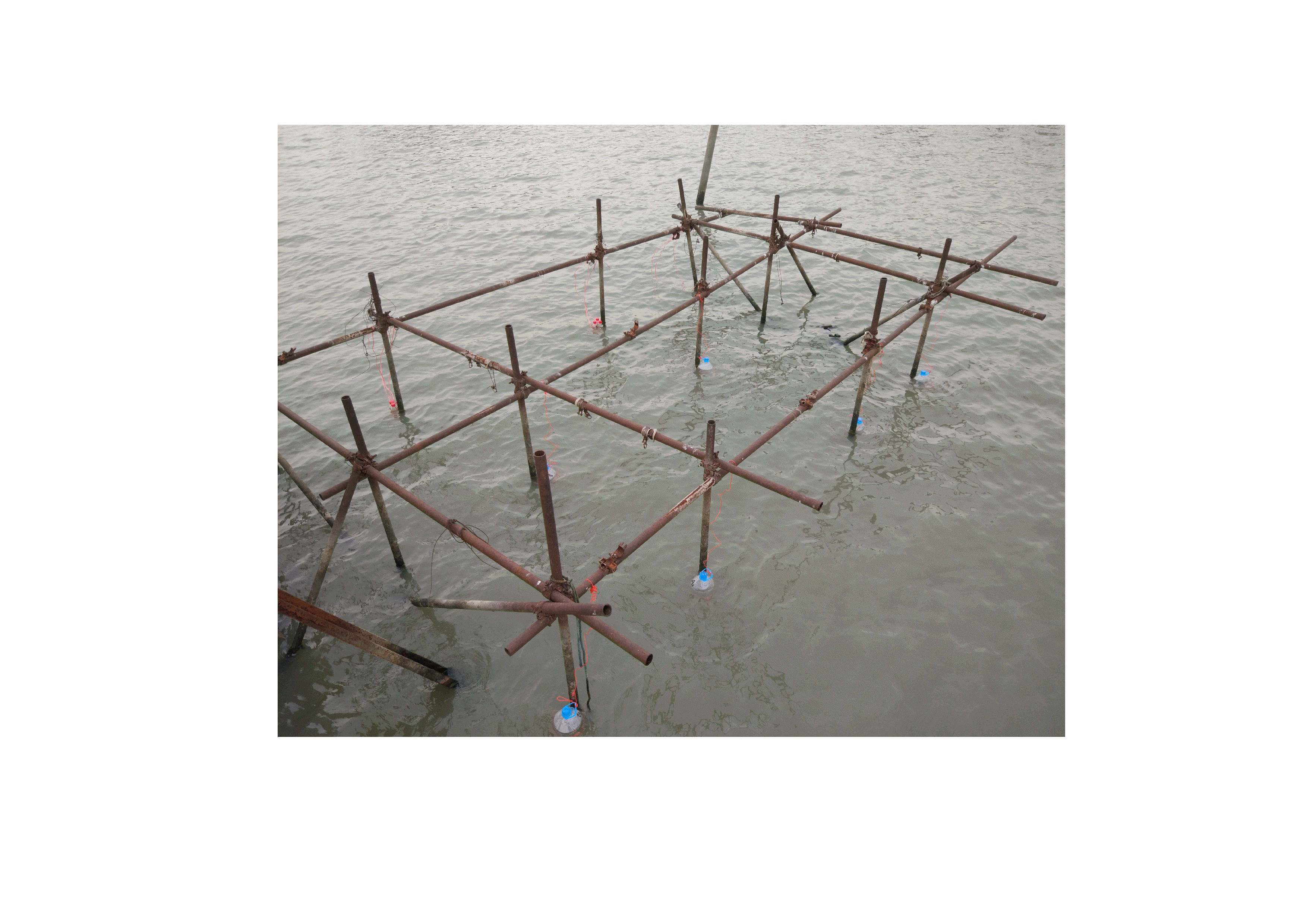

Supplement: FIGURE S2 — The spot picture of in situ experiment of mixing induced by wind-wave on colony size of Microcystis in Lake Taihu. [file Image_2.png]

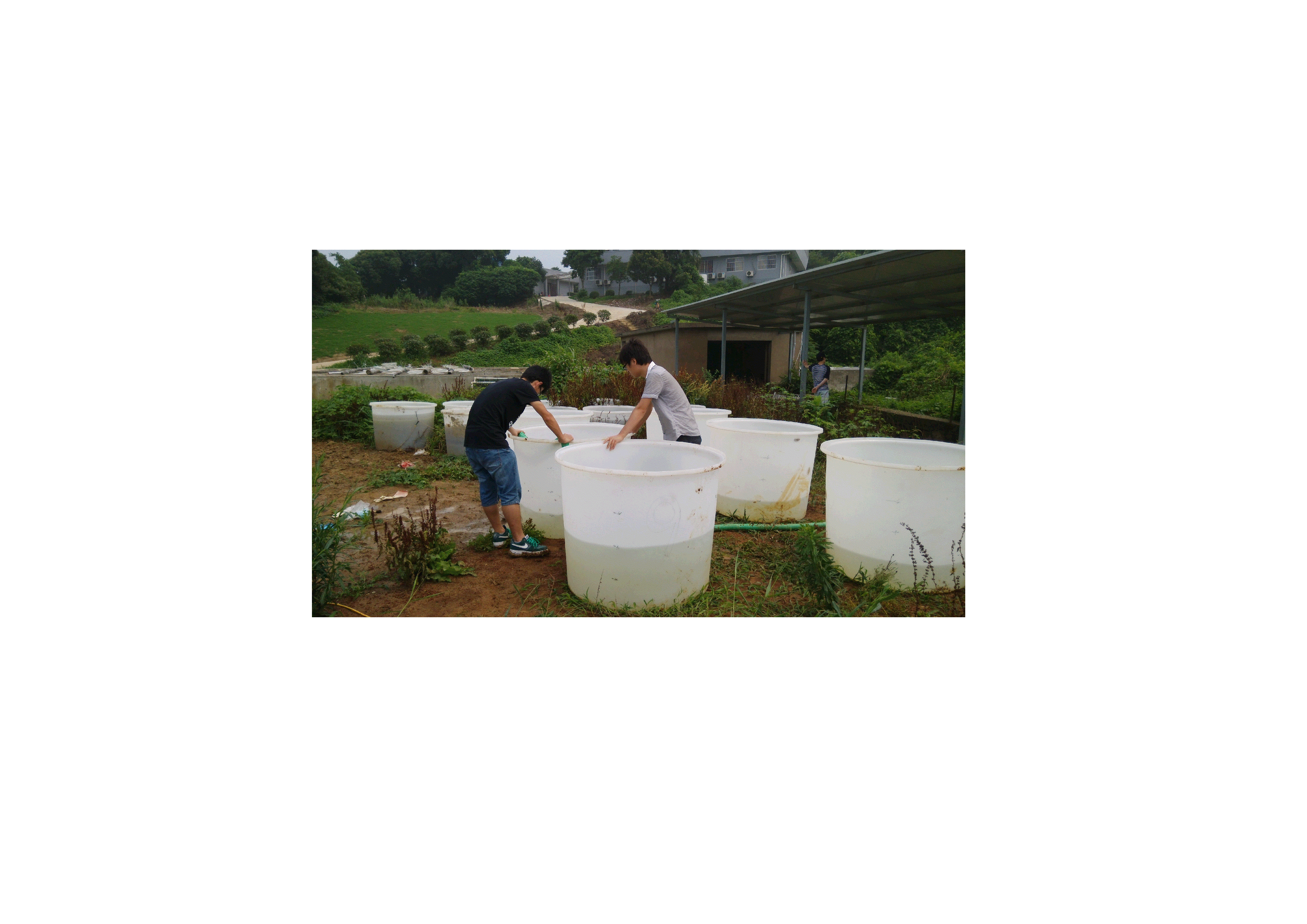

Supplement: FIGURE S3 — The spot picture of in situ simulative mixing on colony size of Microcystis in the Lake Taihu. [file Image_3.png]

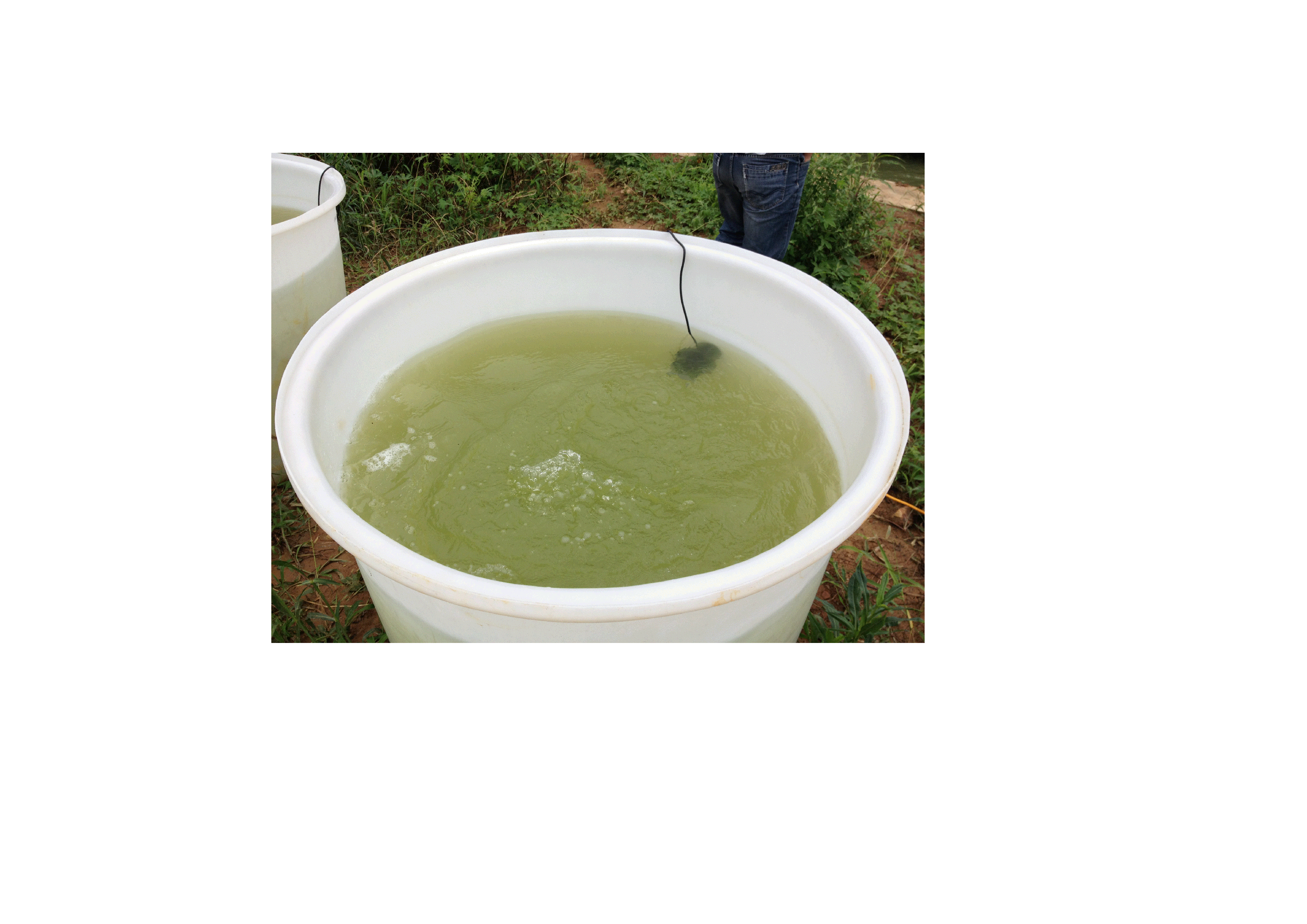

Supplement: FIGURE S4 — The satellite image revealed the cyanobacterial bloom occurrence during post Morak Typhoon. (A) Little bloom with area 92 km2 was detected at 10:27 am, August 12, 2009, when the typhoon peak had passed. (B) Large bloom with area 391 km2 was detected at 11:09, August 13, 2009. [file Image_4.png]

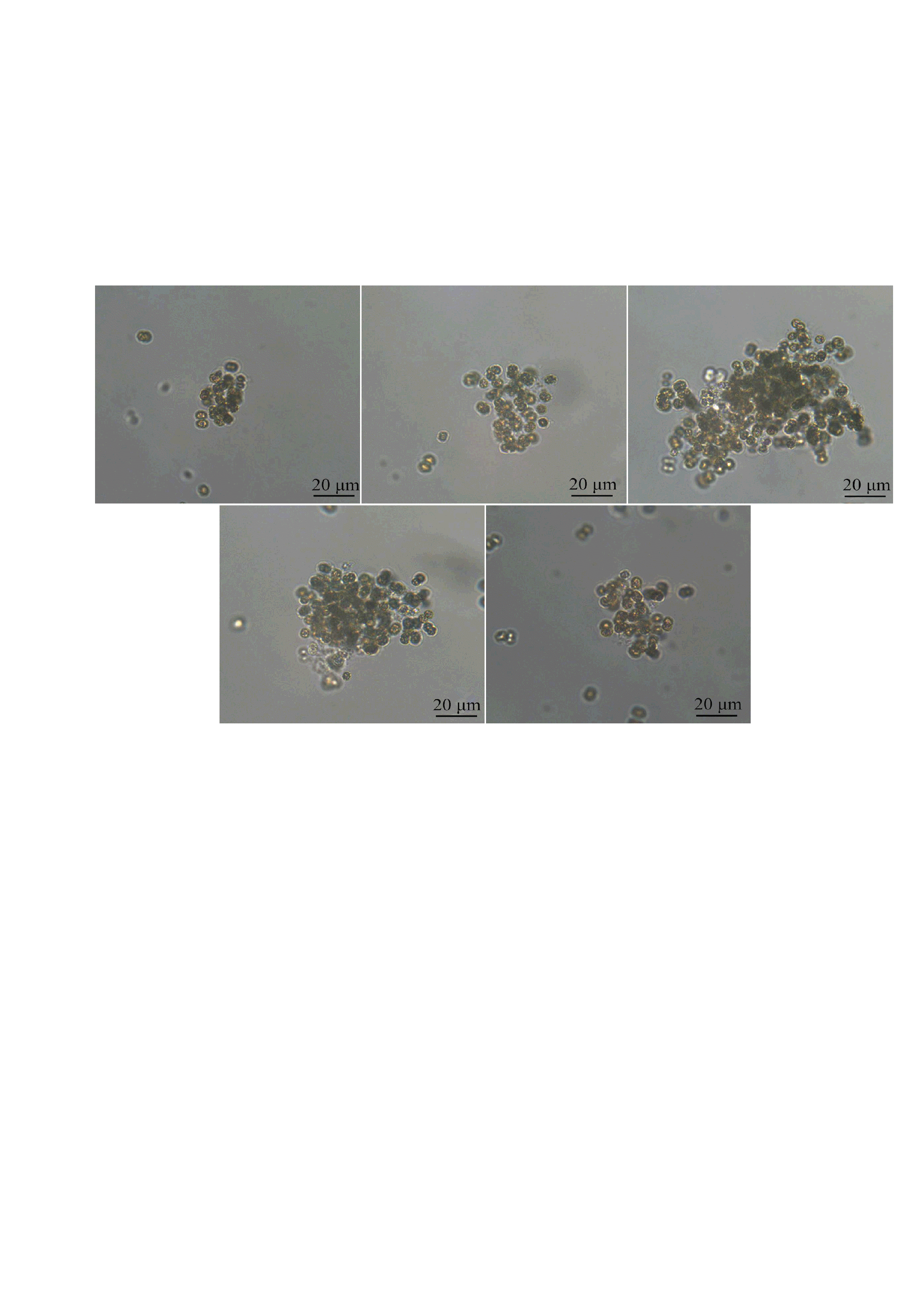

Supplement: FIGURE S5 — The satellite images of cyanobacterial bloom during the Typhoon Soulik passed Lake Taihu in July 2013. The left: image of bloom at 13:10 h, July 12, 2013; the middle: image of bloom at 11:15 h, July 14, 2013; the right: image of bloom at 11:50 h, July 17, 2013. [file Image_5.png]
