# Supplementary material for: Spatiotemporal Changes of Cyanobacterial Bloom in Large Shallow Eutrophic Lake Taihu, China
Source: Front Microbiol. 2018 Mar 21;9:451. doi: 10.3389/fmicb.2018.00451 (PMC5871682; doi:10.3389/fmicb.2018.00451)
Supplement: TABLE S1 — Satellite detected cyanobacterial bloom area changes in a short period in Lake Taihu. [file Table_1.docx]

Table S1 Satellite detected cyanobacterial bloom area changes in a short period in Lake Taihu

| Beginning time | Ending time | Area at beginning (km^2^) | Area at ending (km^2^) |
| --- | --- | --- | --- |
| 2009/5/8 10:28 | 2009/5/8 13:40 | 10.8 | 11.7 |
| 2009/5/9 11:11 | 2009/5/9/12:47 | 11.5 | 16.9 |
| 2009/5/11 10:52 | 2009/5/11 12:36 | 43.8 | 10.3 |
| 2009/5/22 10:40 | 2009/5/22 13:52 | 23.4 | 47.1 |
| 2009/6/12 10:04 | 2009/6/13 13:16 | 11.1 | 366.9 |
| 2009/6/23 10:39 | 2009/6/23 13:52 | 190.4 | 153.9 |
| 2009/7/3 11:16 | 2009/7/3 12:52 | 295.2 | 223.3 |
| 2009/10/26 10:09 | 2009/10/26 13:20 | 139.7 | 53.4 |
| 2013/11/18 13:50 | 2013/11/19 11:15 | 52.8 | 1089.2 |
| 2014/12/1 11:05 | 2014/12/2 10:10 | 109.5 | 600.1 |
| 2014/5/27 10:40 | 2014/5/28 11:15 | 126.2 | 396.4 |
| 2013/4/15 12:15 | 2013/4/17 11:15 | 89.2 | 178.3 |
| 2013/11/15 13:15 | 2013/11/16 10:45 | 920.1 | 142 |
| 2013/5/1 14:00 | 2013/5/2 13:35 | 239.23 | 18.1 |
| 2014/2/10 10:10 | 2014/2/11 10:50 | 209 | 134.6 |
| 2014/7/7 10:40 | 2014/7/8 11:20 | 244.4 | 49.37 |
