# Supplementary material for: Spatiotemporal Changes of Cyanobacterial Bloom in Large Shallow Eutrophic Lake Taihu, China
Source: Front Microbiol. 2018 Mar 21;9:451. doi: 10.3389/fmicb.2018.00451 (PMC5871682; doi:10.3389/fmicb.2018.00451)
Supplement: TABLE S2 — Satellite detected cyanobacterial bloom area changes in a short period in Lake Chaohu. [file Table_2.docx]

Table S2 Satellite detected cyanobacterial bloom area changes in a short period in Lake Chaohu

| Beginning time | Ending time | Area at beginning (km^2^) | Area at ending (km^2^) |
| --- | --- | --- | --- |
| 2015-2-11 10:25 | 2015-2-12 11:00 | 0 | 175.04 |
| 2014-10-25 10:55 | 2014-10-26 13:05 | 53.75 | 196.1 |
| 2014-7-9 10:25 | 2014-7-10 11:10 | 14.9 | 115.1 |
| 2015-4-25 10:15 | 2015-4-26 10:55 | 0 | 54 |
| 2013-9-2 12:45 | 2013-9-3 13:20 | 137.4 | 0 |
| 2014-12-7 10:30 | 2017-12-8 11:15 | 104.81 | 0 |
| 2015-1-19 10:10 | 2015-1-20 11:00 | 85.78 | 9.67 |
| 2013-7-12 13:10 | 2013-7-13 13:15 | 73.6 | 0 |
