# Supplementary material for: Spatiotemporal Changes of Cyanobacterial Bloom in Large Shallow Eutrophic Lake Taihu, China
Source: Front Microbiol. 2018 Mar 21;9:451. doi: 10.3389/fmicb.2018.00451 (PMC5871682; doi:10.3389/fmicb.2018.00451)
Supplement: TABLE S3 — Proportion and floating rate of different colony sizes of Microcystis. [file Table_3.docx]

Table S3 Proportion and floating rate of different colony sizes of *Microcystis*

| Colony size（μm） | >425 | 100-425 | 64-100 | 20-64 | <20 |
| --- | --- | --- | --- | --- | --- |
| Proportion (100%) | 13 | 57 | 13 | 12 | 5 |
|  | 9 | 56 | 16 | 15 | 4 |
|  | 8 | 69 | 11 | 11 | 1 |
| Average | 10 | 61 | 13.3 | 12.7 | 3.3 |
| Variance | 2.6 | 7.2 | 2.5 | 2.1 | 2.1 |
|  |  |  |  |  |  |
| Floating rate (cm/s) | 1.017 | 0.350 | 0.032 | 0.008 | 0.0002 |
|  | 0.700 | 0.210 | 0.020 | 0.012 | 0.0002 |
|  | 0.583 | 0.323 | 0.035 | 0.007 | 0.0002 |
| Average | 0.767 | 0.295 | 0.028 | 0.008 | 0.0002 |
| Variance | 0.225 | 0.075 | 0.008 | 0.003 | 0 |
